# An innovative additively manufactured implant for mandibular injuries: Design and preparation processes based on simulation model

**DOI:** 10.3389/fbioe.2022.1065971

**Published:** 2022-11-24

**Authors:** Lingling Zheng, Chao Wang, Min Hu, Antonio Apicella, Lizhen Wang, Ming Zhang, Yubo Fan

**Affiliations:** ^1^ Key Laboratory of Biomechanics and Mechanobiology, Ministry of Education, Beijing Advanced Innovation Center for Biomedical Engineering, School of Biological Science and Medical Engineering, School of Engineering Medicine, Beihang University, Beijing, China; ^2^ The First Medical Center of PLA General Hospital, Department of Stomatology, Beijing, China; ^3^ Polytechnique School of Engineering and Base Science, University of Campania, Aversa, CE, Italy; ^4^ Department of Biomedical Engineering, The Hong Kong Polytechnic University, Kowloon, Hong Kong SAR, China

**Keywords:** large bone defects, mandibular injury, lattice-like implant, bone regeneration, biomechanics and mechanobiology

## Abstract

**Objective:** For mandibular injury, how to utilize 3D implants with novel structures to promote the reconstruction of large mandibular bone defect is the major focus of clinical and basic research. This study proposed a novel 3D titanium lattice-like implant for mandibular injuries based on simulation model, which is designed and optimized by a biomechanical/mechanobiological approach, and the working framework for optimal design and preparation processes of the implant has been validated to tailored to specific patient biomechanical, physiological and clinical requirements.

**Methods:** This objective has been achieved by matching and assembling different morphologies of a lattice-like implant mimicking cancellous and cortical bone morphologies and properties, namely, an internal spongy trabecular-like structure that can be filled with bone graft materials and an external grid-like structure that can ensure the mechanical bearing capacity. Finite element analysis has been applied to evaluate the stress/strain distribution of the implant and bone graft materials under physiological loading conditions to determine whether and where the implant needs to be optimized. A topological optimization approach was employed to improve biomechanical and mechanobiological properties by adjusting the overall/local structural design of the implant.

**Results:** The computational results demonstrated that, on average, values of the maximum von-Mises stress in the implant model nodes could be decreased by 43.14% and that the percentage of optimal physiological strains in the bone graft materials can be increased from 35.79 to 93.36% since early regeneration stages. Metal additive manufacturing technology was adopted to prepare the 3D lattice-like implant to verify its feasibility for fabrication. Following the working framework proposed in this study, the well-designed customized implants have both excellent biomechanical and mechanobiological properties, avoiding mechanical failure and providing sufficient biomechanical stimuli to promote new bone regeneration.

**Conclusion:** This study is expected to provide a scientific and feasible clinical strategy for repairing large injuries of mandibular bone defects by offering new insights into design criteria for regenerative implants.

## Introduction

The mandible is a critical component of the skull maxillofacial bones that have a pivotal role in aesthetic and structural functions such as mastication, swallowing and phonation. Traumas, osteoradionecrosis, malformation, dysplastic pathologies, and benign or malignant neoplasm can cause large mandibular injuries (Torroni et al., 2015; Kumar et al., 2016). Mandibular reconstruction is not only to recover the external contour of maxillofacial region, but also to restore the normal physiological function of the patient. However, reconstruction of large mandibular injuries is still a major challenge in maxillofacial surgery (Vidal et al., 2020).

Although there are many clinical solutions for mandibular reconstruction, all of them have limitations or deficiencies. Firstly, autogenous bone grafts are regarded as the “golden standard” in reconstructive surgery because of its osteoinductive, osteoconductive and non-immunogenic properties (Cypher and Grossman, 1996). Even though several successful clinical results have been reported (Cordeiro et al., 1999; Okay et al., 2016; Brown et al., 2017), the limitation of autografts, such as insufficient donor-site bone material, postoperative infection, and various potential complications, remains a considerable clinical concern (Ahlmann et al., 2002). Secondly, reconstruction of mandible with titanium plate instead of autologous bone can obtain satisfactory aesthetic effect without complications of donor site (Goh et al., 2008; Sadr-Eshkevari et al., 2013). Nevertheless, problems such as extraoral exposure, plate fracture, osteomyelitis and the inability to restore the occlusal function remain major defects of titanium plate (Okura et al., 2005). Thirdly, an alternative solution is allograft (Carter, 1999) and xenograft substitutes (Giannoudis et al., 2005), which can repair local bone defects, such as alveolar bone (Elgali et al., 2017) and/or periodontal tissue (Rasperini et al., 2015). While in large bone defects, the lack of mechanical strength under physiological load is the main drawback. In a word, given the limitations of the above solutions, an innovative solution with biomimetic structure and function needs to be developed to meet the clinical needs of repairing large mandibular injuries.

**FIGURE 1:**
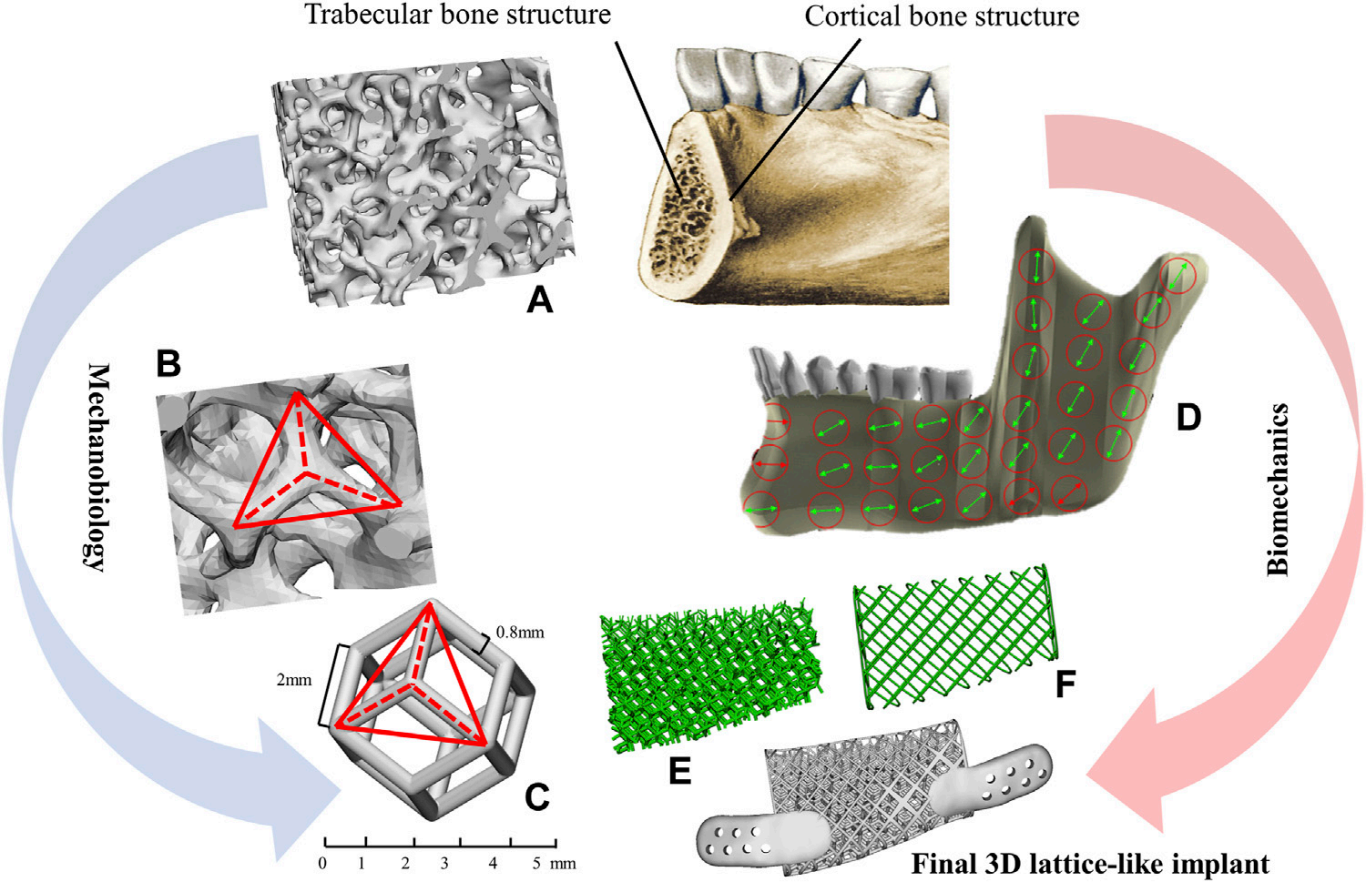
Methodological approach for the lattice-like implants. **(A)** The morphology of trabecular bone. **(B)** The texture characteristics of trabecular. **(C)** A dodecahedral cellular unit. **(D)** The overall view of local orientation of mandible and the arrow indicates the direction of maximum stiffness concerning the occlusal plane. **(E)** Trabecular-like component to mimic the cancellous bone of the mandible. **(F)** Grid-like component to mimic cortical bone of the mandible.

In this study, a novel 3D titanium lattice-like implant with structural design and preparation processes based on simulation model was proposed, which can be filled with bone graft materials to promote bone regeneration. A successful maxillofacial implant would restore the mandibular function and promote bone tissue regeneration at the damaged site. While the biomechanical and mechanobiological processes of bone regeneration are complex (Guilak et al., 2014), thus, more stringent and unique requirements are put forward for the design and manufacture of 3D titanium implants (Figure 1). From the biomechanical standpoint, the implant is a fundamental device for bone regeneration with a continuously space maintenance capacity; the proper mechanical properties and structural parameters choices are critical. The elastic modulus, strength, fatigue and other mechanical properties of customized implants should be up to standard to avoid stress shielding and mechanical failures. Structural parameters, as well as trabecular-like textures, porosity, pore size and pore interconnectivity, are key factors that will significantly influence the biomechanical properties of implants such as bone ingrowth and cell regeneration. From the mechanobiological standpoint, bone regeneration is known to be highly dependent on the local conditions, and mechanobiology studies show how biomechanical stimuli influence the growth and regeneration of bone tissues. Implants providing a complex micro-environment should have mechanobiological stimulation abilities, which can promote the proliferation and differentiation of osteoblasts while guiding bone regeneration. It is reported that implant with different porosities, pore sizes, and strut diameters has different biomechanical stimuli on bone growth and adjusting these structure features can achieve desired bone regeneration outcomes (Sanz-Herrera et al., 2010; Razi et al., 2012; Wang et al., 2016b; Gao et al., 2019). Regretfully, despite the advances in theoretical basis of mandibular reconstruction, comprehensive research and validation studies of 3D titanium implant design and optimization criteria based on biomechanical/mechanobiology methods are still lacking.

Therefore, the aim of this study was to propose a novel 3D titanium lattice-like implant for mandibular injuries based on simulation model, which is designed and optimized by a biomechanical/mechanobiological approach, and validate the working framework for optimal design and preparation processes of the 3D titanium lattice-like implant. This study details the design concept, analysis method, optimization criterion, and manufacturing processes, expected to provide a scientific and feasible clinical solution for reconstructing large mandibular injuries. It is hoped that it will have great clinical application prospect in maxillofacial surgery.

## Materials and methods

**FIGURE 2:**
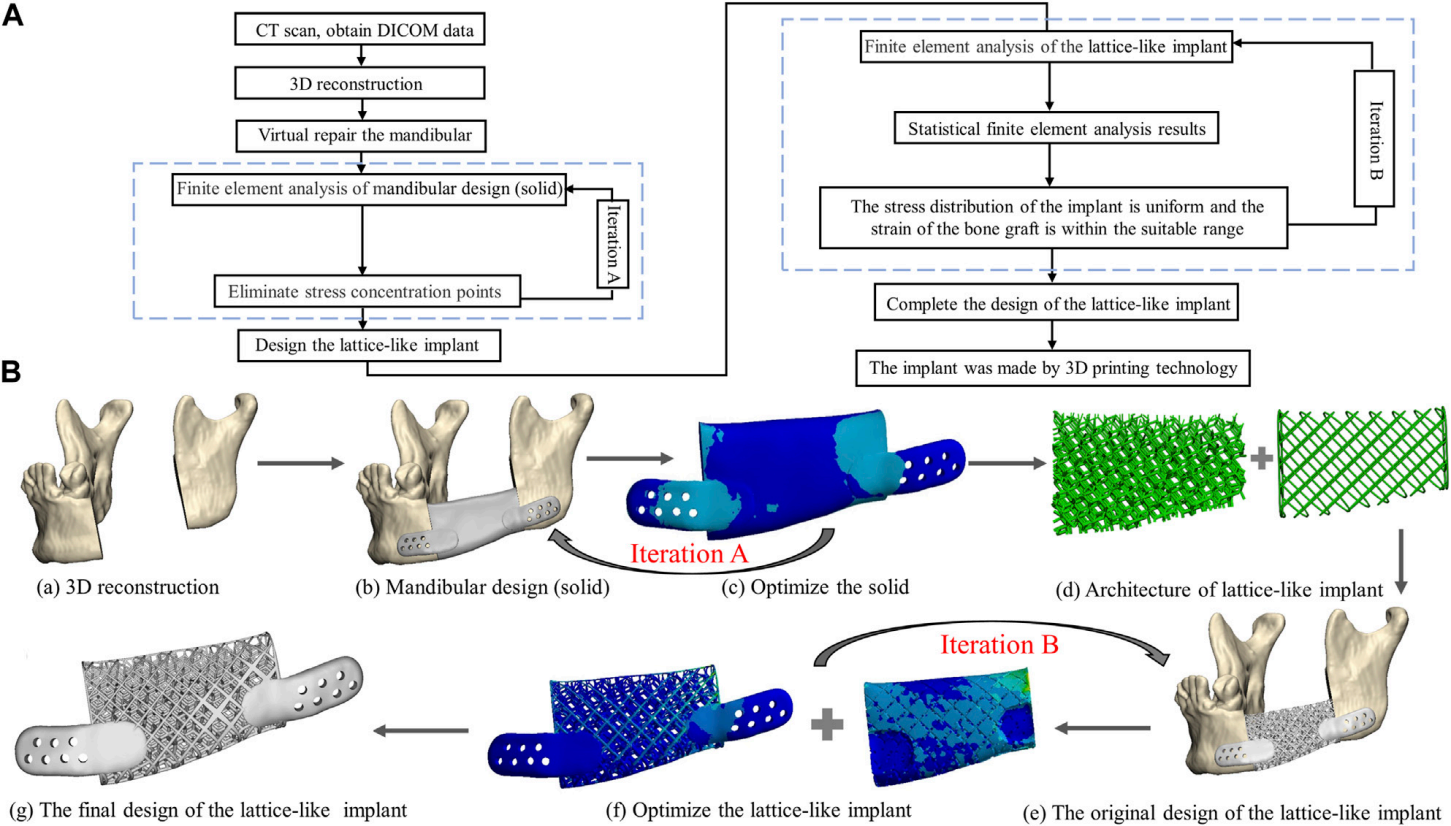
The framework from image acquisition to manufacturing implant. **(A)** Flowchart of lattice-like implant design, optimization and manufacture. **(B)** Schematic diagram of lattice-like implant design, optimization and manufacture. (a) 3D reconstruction. (b) Mandibular design (solid). (c) Optimize the solid counterpart. (d) The architecture of lattice-like implant. (e) The original design of the lattice-like implant. (f) Optimize the lattice-like implant. (g) The final design of the 3D implant.

In this study, the design workflow of the 3D lattice-like implant is shown as follows (Figure 2A):

1) The cone-beam computed tomography (CBCT) data acquisition and three-dimensional (3D) reconstruction of the mandible;
2) Design a solid model for the virtual mandibular reconstruction based on the mirroring reconstruction approach;
3) Optimization iteration of the solid model;
4) Design the implant with the internal trabecular-like structure and the external grid-like structure;
5) Optimization iteration of the lattice-like implant based on biomechanical strength and mechanobiological critical levels;
6) Additive manufacturing technologies adopted to fabricate the optimized lattice-like implant.

More details are given in the next sections.

### 3D modelling reconstruction

**FIGURE 3:**
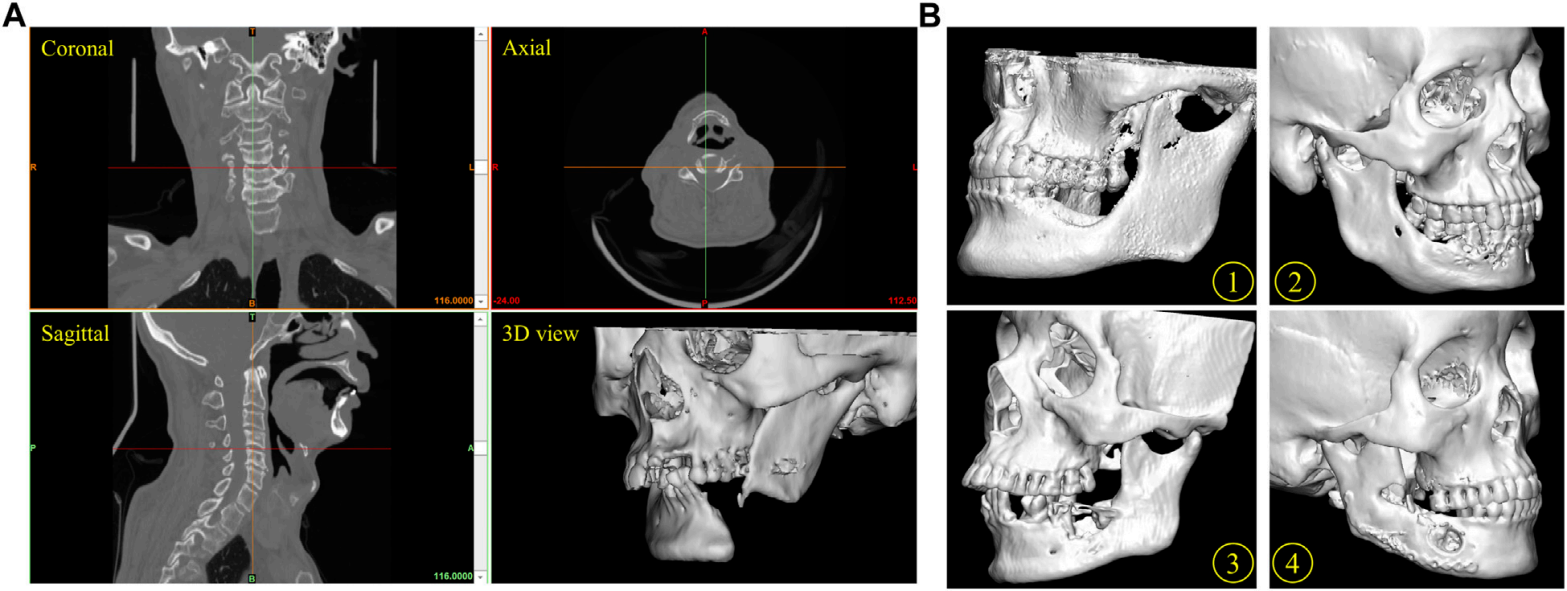
Reconstruction of mandible defect based on CBCT image. **(A)** The process for the generation of a three-dimensional (3D) mandibular reconstruction. Coronal view, axial view, sagittal view and 3D model of mandibular. **(B)** Several mandibular defects data were used in this study. Patients 1, 2, 3 and 4.

The cone-beam computed tomography (CBCT) data acquisition needed for the reconstruction of the missing mandible section was obtained from the Department of Oral and Maxillofacial Radiology with the following parameters: slice thickness 0.4 mm, pixel size 0.4 mm. The cone-beam computed tomography (CBCT) images were imported into Mimics software (Mimics Innovation Suite v.21.0, Materialise, Leuven, Belgium). Software inbuilt specific craniofacial bone region growing greyscales threshold values of the Hounsfield units (HU) were selected to define the bounds and properties of the solid parts. Then a 3D digital model of the mandible was generated and saved in the standard tessellation language file format (.STL), which served as the basis for the computer aided design (CAD) of mandible geometry. Figure 3 shows the process for generating a 3D volumetric mandibular reconstruction and the mandibular defects data investigated in this study.

### Design process of the lattice-like implant

The patient mandible 3D model was imported in STL format into the medically certified computer aided design (CAD) modelling software 3-Matic 12.0 (Materialise, Leuven, Belgium). Prior to the implant lattices definition, it was first needed to anatomically restore the mandible defect using a reconstruction approach mirroring and fitting the symmetrical healthy mandible section in the space of its missing part (Figure 2Ba). Then, the general implant structure was outlined and discussed with the surgeon for appropriate length, width, and height (Figures 2Bb,c).

The typical mandibular structure with cortical and trabecular bone is the methodological base approach for designing biomimetic 3D mandibular reconstruction implant. The lattice textures mimicking cancellous and cortical bone morphologies and properties were then chosen (Figure 2Bd). Specifically, the lattice-like implant comprised two types: internal spongy interconnected trabecular-like structure and external grid-like structure mimicking cancellous and cortical bone morphologies and properties, respectively.

*Cancellous bone component*- The structure of cancellous bone is composed of thin trabecular which are the natural evolutions of functional orientations corresponding to tension and compression lines induced by bone loading from teeth and muscles. The regular dodecahedron structure (Figures 1B,C) has been suggested as representative of 3D organization of trabecular bone from previous studies (Gramanzini et al., 2016; Ben-Zvi et al., 2017). Therefore, to mimic the same biomechanical environment that describes the cancellous bone component of the mandible, regular dodecahedron cellular was utilized to construct the trabecular-like component of implant. It is worth noting that, in order to facilitate the intraoperative filling autogenous bone particles or bone graft materials according to the clinical practice and literature (Gao et al., 2019), the pore diameter of implant is set at 5 mm.

*Cortical bone component* -The cortical bone structural texture was designed as a quadrilateral grid with the similar pore diameter to construct the grid-like component of implant. In addition, the strength of mandible gradually increases from the top of alveolar crest to the lower edge, and the implant stress gradually increases from top to bottom (Gao et al., 2019). Therefore, the lattice-like implant was designed with a gradient structure where the lower and upper limits of the strut diameter were set, respectively, to 0.2 and 0.8 mm to avoid stress shielding while ensuring the necessary mechanical strength. And the thickness of the retainer is 2 mm, and there is 5–7 titanium screws on each side (Figure 2B).

### Finite element analysis of the lattice-like implants

Finite element analysis (FEA) was adopted to evaluate the stress of solid/implant structure and strain distributions within bone graft materials under physiological loading condition. The bone graft model was derived from Boolean subtraction of the solid models of the mandible and lattice-like implant. The screws were designed as cylinders of 8 mm in length. 3D models were imported into ANSYS 19.0 software (Swanson Analysis System Co, Houston, TX, United States) for the FE calculation. The number of the elements and nodes is shown in Table 1.

**TABLE 1:** Numbers of elements and nodes and mechanical properties.

Materials	Nodes	Elements
Cortical bone	47,407	185,457
Cancellous bone	13,277	45,379
Tooth	10,832	35,464
Articular disc	6,008	20,219
Lattice-like implant	299,536	904,451
Bone graft materials	77,195	306,956
Screws	8,227	27,434
Materials	Young’s modulus [MPa]	Poisson’s ratio
Cortical bone Sarrafpour et al. (2013)	15,000	0.3
Cancellous bone Sarrafpour et al. (2013)	1,500	0.3
Articular disc Sarrafpour et al. (2013)	44.1	0.4
Tooth Sarrafpour et al. (2013)	20,000	0.3
Ti6Al4V Manivasagam et al. (2009)	110,000	0.3
Granulation tissue Checa et al. (2011)	0.2	0.167
Immature bone Checa et al. (2011)	1,000	0.3
Mature bone Checa et al. (2011)	5,000	0.3

All materials were considered isotropic, homogeneous and linearly elastic. The load transfer capacity of the implant filled with bone graft materials at different stages of the healing time was evaluated by associating literature grafted bone mechanical properties data to 3 different periods of bone regeneration: Granulation tissue (G-T) representing the healing early stages, Immature bone (IM-B) representing the middle period, and mature bone (M-B) representing the final period (Checa et al., 2011). The mechanical properties used in this study were taken from literature data for cortical bone, cancellous bone, articular disc, tooth, Ti6Al4V and grafted bone at different stages of maturation (Manivasagam et al., 2009; Checa et al., 2011; Sarrafpour et al., 2013), as reported in Table 1.

**FIGURE 4:**
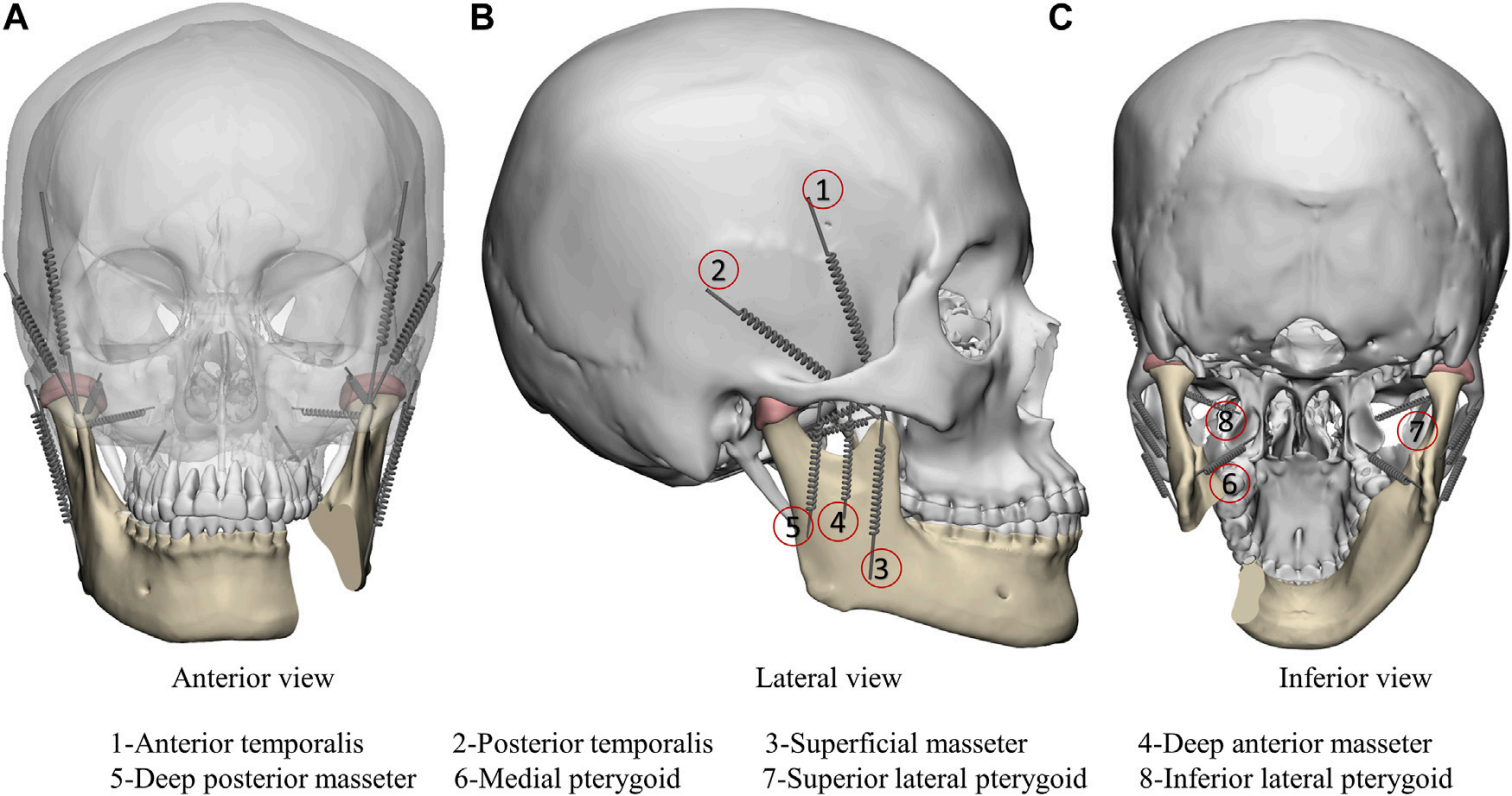
Finite element model boundary conditions including anterior view **(A)**, lateral view **(B)** and inferior view **(C)**. Masticatory muscle was simulated as spring element: 1-Anterior temporalis; 2-Posterior temporalis; 3-Superficial masseter; 4-Deep anterior masseter; 5-Deep posterior masseter; 6-Medial pterygoid; 7-Superior lateral pterygoid; 8-Inferior lateral pterygoid.

In this study, simulating the mandibular movement controlled by masticatory muscles under maximum bite force (worse case) with 800 N (Ackland et al., 2017) and eight groups of masticatory muscles were simulated as spring element and set on each side (Figure 4). The muscle force value and the direction of the muscle structures were taken from the published research (Ackland et al., 2017) (Table 2). The springs’ stiffness values are as follows: temporalis muscle = 14 N/mm, masseter muscle = 16.35 N/mm, lateral pterygoid muscle = 12 N/mm, and medial pterygoid muscle = 15 N/mm.

**TABLE 2:** Muscle force generated under maximum-force bite.

		Maximum bite force (800N)
Left	Anterior temporalis	222.3
	Posterior temporalis	158.6
	Superior lateral pterygoid	5.1
	Inferior lateral pterygoid	65.5
	Medial pterygoid	170.8
	Superficial masseter	196.3
	Deep anterior masseter	37.9
	Deep posterior masseter	44.9
Right	Anterior temporalis	221.5
	Posterior temporalis	156.5
	Superior lateral pterygoid	5.3
	Inferior lateral pterygoid	76.1
	Medial pterygoid	170.7
	Superficial masseter	196.3
	Deep anterior masseter	37.9
	Deep posterior masseter	44.2

### Optimization iteration of the lattice-like topological structure

The Finite Element Analyses under physiological load involves two stages of iterative process in this study. Iteration A represents solid optimization aimed at eliminating stress concentration points and thin-walled structures between the implant and the residual bone, and determining the number and location of titanium screws. Iteration B represents lattice-like implant optimization, which considers the biomechanical properties of the implant and the mechanobiological factors of bone grafts. The lattice-like implant needs to be optimized or not was determined by the FEA results, both stress of implant and strain of bone graft material. The implant ought to possess sufficient mechanical properties to avoid mechanical failure while inducing in the bone graft material optimal physiological strain levels stimulating bone regeneration. That is, the maximum von-Mises stress of the implant cannot exceed 897 MPa of the yield strength of Ti6Al4V (Manivasagam et al., 2009), and the strain adaptation ranges of bone graft material remodeling is 0–3,000 microstrain (Frost, 2004). The original designed lattice-like implant needed to be adjusted until it meets above criterion based on the FEA results, which is considered an optimizing accomplish of the topological structure.

### Preparation for implants by additive manufacturing

Additive manufacturing (AM) technologies have been adopted to fabricate titanium implants with complex structures. The model was imported into QuantAM software (Renishaw, Gloucestershire, England) for adding supports, slicing and developing an “mtt” format file to be transferred to the additive manufacturing machine. RenAM400 (Renishaw, Gloucestershire, England) equipped with SPI continuous fibre laser 200 W, optical maser wavelength 1,075 nm, spot diameter 50–80 µm, forming layer thickness 20–50 μm, focal length 163 mm, galvanoscope laser scanning with scanning speed 5–7,000 mm/s and the working chamber is protected by argon gas.

## Results

### Finite element analysis results of original non-optimized 3D lattice-like implant

**FIGURE 5:**
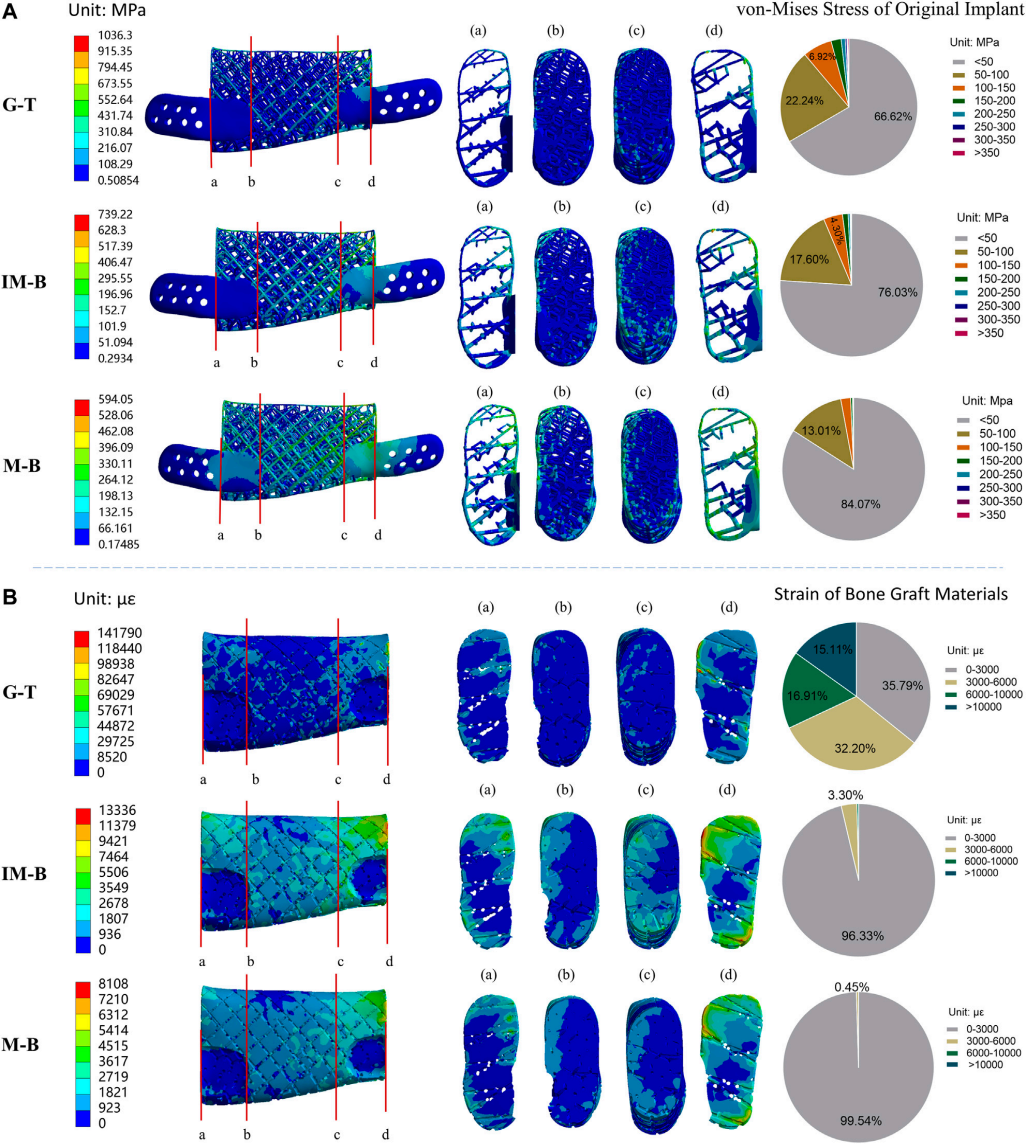
Von-Mises stress of original lattice-like implant and strain of bone graft materials in different periods. Granulation tissue (G-T) representing the early stages, Immature bone (IM-B) representing the middle period and mature bone (M-B) representing the final period. **(A)** The von-Mises stress of original implant in different periods. **(B)** The strain of bone graft materials in different periods. **(a–d)** illustrated four sections of von-Mises stress/strain for the original implant/bone graft materials. The pie charts show the frequency of von-Mises stress/strain for the original implant/bone graft materials in different intervals.

**FIGURE 6:**
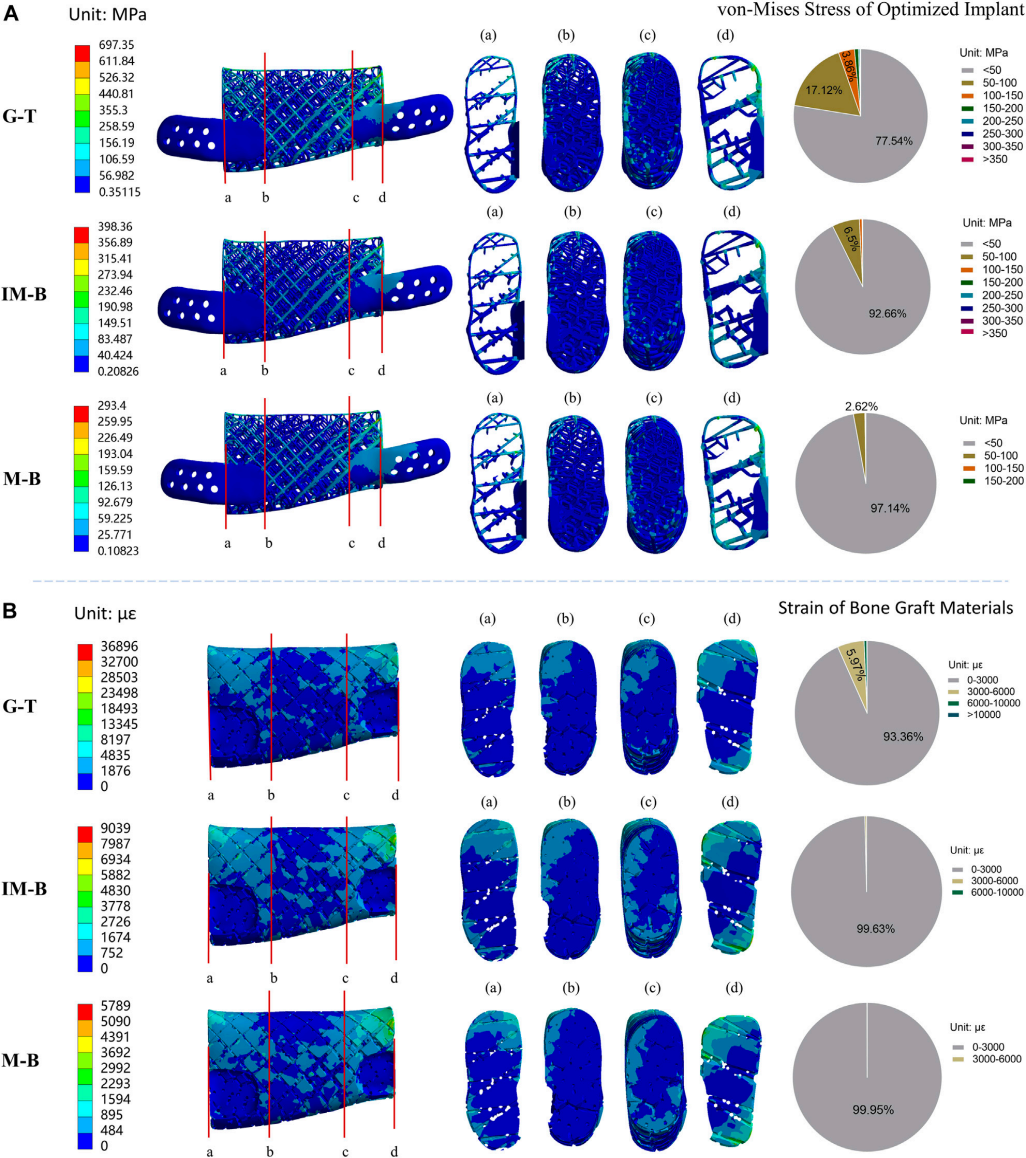
Von-Mises stress of optimized lattice-like implant and strain of bone graft materials in different periods. Granulation tissue (G-T) representing the early stages, Immature bone (IM-B) representing the middle period and mature bone (M-B) representing the final period. **(A)** The von-Mises stress of optimized implant in different periods. **(B)** The strain of bone graft materials in different periods. **(a–d)** illustrated four sections of von-Mises stress/strain for the original implant/bone graft materials. The pie charts show the frequency of von-Mises stress/strain for the original implant/bone graft materials in different intervals.

The finite element analysis results of the physiologically loaded starting non-optimized and final optimized implants at the three hypothesized healing times (G-T, IM-B and M-B) are shown in Figure 5 and Figure 6, respectively. Frequency analysis was used to statistics analyze the stress of implant and strain distribution of bone graft materials (pie charts in Figures 5, 6). Namely, eight stress intervals (<50, 50–100, 100–150, 150–200, 200–250, 250–300, 300–350, >350 MPa) and four strain intervals (0–3,000, 3,000–6,000, 6,000–10,000,>10,000 με) were considered. The maximum von-Mises stress values reached in the non-optimized initial lattice-like implant are reported in Figure 5A, and are 1,036.30, 739.22 and 594.05 MPa for G-T, IM-B and M-B periods, respectively. The corresponding maximum strain values reached in the bone grafts are 1,41,790, 13,336 and 8,108 με for G-T, IM-B and M-B periods, respectively (Figure 5B).

*Stress distribution of implant at G-T, IM-B and M-B healing times*- In the first granulation tissue (G-T) healing period, the 66.62% of the von-Mises stress values in the nodes of the implant were lower than 50 MPa, while the 22.24% fall in the 50–100 MPa range, the 6.92% are located between 100 and 150 MPa, and the remaining 4.22% overcome 150 MPa. In the immature bone (IM-B) period, the 76.03% of the von-Mises stress values in the nodes of the implant were lower than 50 MPa, while the 17.60% fall in the 50–100 MPa range, the 4.30% are located between 100 and 150 MPa, and the remaining 2.07% overcome 150 MPa. In presence of mature bone (M-B), the 84.07% of the von-Mises stress values in the nodes of the implant were lower than 50 MPa, while the 13.01% fell in the 50–100 MPa range, and only a 3% were above 100 MPa.

*Strain distribution of bone graft at G-T*, *IM-B and M-B healing times*- In the first granulation tissue (G-T) healing period, only the 35.79% of the strain values are lower than the maximum physiological strain of 3,000 με, while the 32.20% are in the 3,000–6,000 με range, the 16.91% are in the 6,000–10,000 με range, and 15.11% are higher than 10,000 με. In the immature bone (IM-B) healing time, the 96.33% of the strain values present strains lower than the critical 3,000 με level. The percentage of healthy physiological strain levels reached the 99.54% of the nodes in presence of mature bone (M-B).

### Finite element analysis results for the optimized 3D lattice-like implant

The finite element analysis results of the optimized lattice-like implant at three different healing periods under physiological load conditions are shown in Figure 6. The values of the maximum von-Mises stress reached in the optimized implant are reported in Figure 6A, and are 697.35, 398.36 and 293.40 MPa for G-T, IM-B and M-B periods, respectively. The corresponding maximum strain values reached in the bone grafts are shown in Figure 6B, and are 36,896, 9,039 and 5,789 με for G-T, IM-B and M-B periods, respectively.

*Stress distribution of implant at G-T, IM-B and M-B healing times*- In the granulation tissue (G-T) healing period, the 77.54% of the von-Mises stress values in the nodes of the implant were lower than 50 MPa, while the 17.12% fall in the 50–100 MPa range, the 3.86% are located between 100 and 150 MPa, and the remaining 1.48% overcome 150 MPa. In the immature bone (IM-B) period, the 92.66% of the von-Mises stress values in the nodes of the implant were lower than 50 MPa, while the 6.50% fall in the 50–100 MPa range, and only a 0.84% were above 100 MPa. In presence of mature bone (M-B), the 97.14% of the von-Mises stress values in the nodes of the implant were lower than 50 MPa, while the 2.62% fall in the 50–100 MPa range, and only a 0.24% were above 100 MPa.

*Strain distribution of bone graft at G-T, IM-B and M-B healing times*- In the granulation tissue (G-T) healing period, the strain values of 93.36% in the nodes are lower than the maximum physiological strain of 3,000 με, while the 5.97% are in the 3,000–6,000 με range, and 0.67% are higher than 6,000 με. In the immature bone (IM-B) healing time, the 99.63% in the nodes of the strain values present strains lower than the critical 3,000 με level. The percentage of healthy physiological strain levels reached the 99.95% of the nodes in presence of mature bone (M-B).

The results indicated that the maximum von-Mises stress of the support did not exceed the yield strength of titanium and almost throughout the bone graft materials received the appropriate strain (0–3,000 με) to stimulate bone regeneration from the early implantation stages.

### Optimization process and results

**FIGURE 7:**
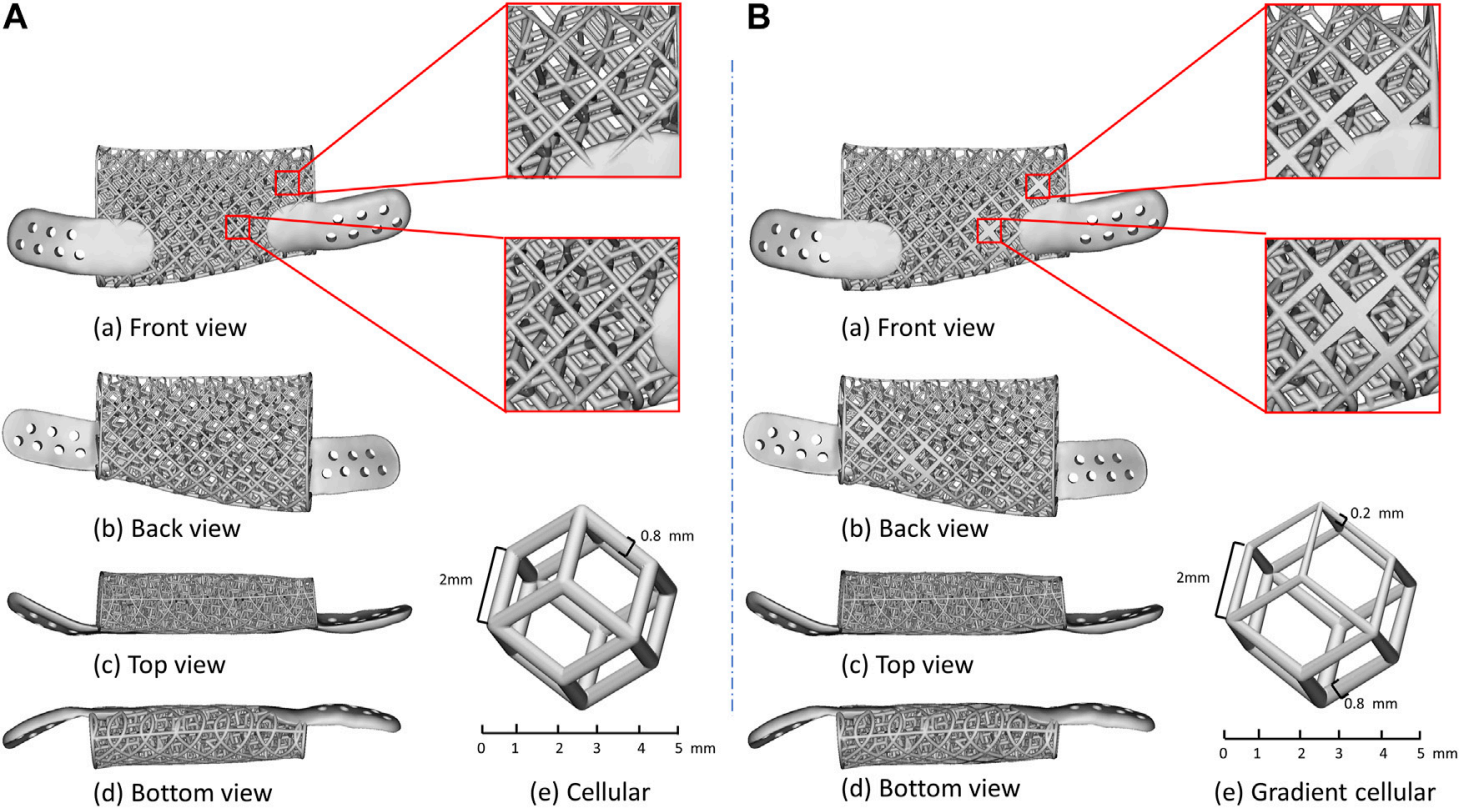
Details of lattice-like implant comparison. **(A)** Original lattice-like implant with a front view (a), back view (b), top view (c), bottom view (d) and regular dodecahedron unit cellular (e); **(B)** Optimized lattice-like implant with a front view (a), back view (b), top view (c), bottom view (d) and gradient cellular (e).

The finite element analysis results demonstrated that the maximum von-Mises stress values of the implants and the strain percentage of bone graft varied obviously during the optimization process. The section color stress maps of the finite element model indicate that the internal stress values of the initial lattice-like implant are generally less than 100 MPa, and the stress concentration region is mainly on the distal surface. Hence, there are warranted to optimize the stress concentration area by tuning the local struts diameters on the distal surface, and the comparison diagram of implant optimization is shown in Figure 7. Compared to the initial implant, the optimized implant achieved a 43.14% average reduction in maximum von-Mises stress, and the adaptation strain levels of bone graft already increased from 35.79% to 93.36% in the early regenerative stages. The results manifested that the optimization process was highly effective.

### 3D additive manufacturing implant

**FIGURE 8:**
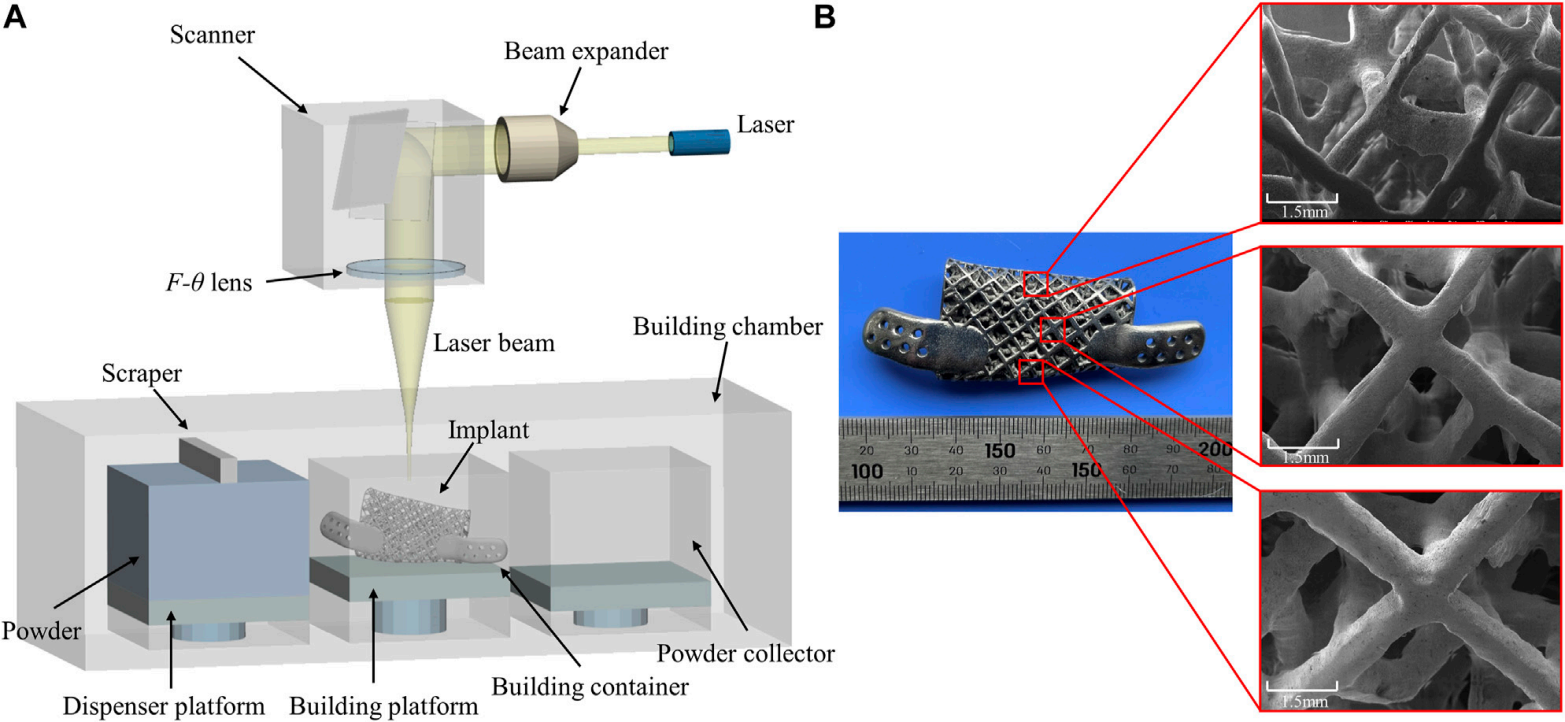
**(A)** Schematic diagram of Selective Laser Melting technology and **(B)** additively manufactured implant with scanning electron micrographs of different locations.

The lattice-like implant was fabricated by Selective Laser Melting (SLM) and using the commercially Ti6Al4V medical-grade powder with an average diameter of 40 μm. The galvanometer laser scanning system directs the laser beam to melt the powder and shape it layer by layer until the implant is completed (Figure 8A). After that, heat treatment in argon-protected was used for the implant, heating the furnace to 820°C within 4 h and cooling it to 500°C within 1.5 h. Subsequent post-processing procedures include sandblasting with a particle size of 0.5–1 mm silicon sand, grinding and polishing the implant surface and ultrasonic cleaning with ultrapure water for 30 min. Finally, the implant was were photographed by scanning electron microscope. The 3D additive manufacturing schematic diagram and the finished implant with electron micrograph are shown in Figure 8B.

## Discussion

The goals and the criteria for a successful reconstruction of critically injuries of mandibular are to restore facial contours, bone regeneration and occlusal reconstruction, which remains a challenge for maxillofacial surgery today (Lin et al., 2011). Although there are several options for mandibular reconstructions, such as autograft, allograft and titanium plate repairs, efforts are being made to develop a new biomimetic design and manufacturing methods for mandibular implants remain underway. With the rapid development and popularization of computer technology, finite element analysis, a numerical method to simulate the modelling of structures that approximates reality, has been widely used in almost all scientific fields (Boccaccio et al., 2011). And with the development of 3D additive manufacturing techniques, selective laser melting (SLM) technology has been used to fabricate customized implants with complicated architectonics, which is regarded as one of the most promising techniques to be associated with medical imaging (Rengier et al., 2010). The improvement in technologies is increasingly leveraging the innovation in reconstructing large segmented defects of mandibular.

Many studies have focused on customized implants designed *via* finite element methods and produced by additive manufacturing over the past decades. Earlier, Lee and Tideman (Lee et al., 2008; Lee et al., 2009) proposed the concept of a modular endoprosthesis for mandibular reconstruction and conducted a series study on Macaca monkeys. Whereas that approach was ineffective and suffered from infection, prosthesis connections loosen and loss of peri-implant bone mineral density. Subsequently, the patient-specific titanium plates utilized to reconstruct maxillofacial bone defects were of great interest. Lim et al. (2022) conducted a long-term follow-up of 16 patients who underwent reconstruction using patient-specific titanium plates. Albeit this customized-plates reduced fixation failure and aesthetically unsatisfactory complications, it cannot rehabilitate the masticatory function, which remains a deficiency. Another possible solution to address aesthetic and functional clinical demands is to produce 3D titanium mesh implants using additive manufacturing. A research institute (Pobloth et al., 2018; Perier-Metz et al., 2020) investigated that mechanobiological optimized 3D titanium mesh implants can promote endogenous bone regeneration based on the implantation of titanium implants with autologous bone graft into the long bones of 27 sheep. After that, they explored the existing mechano-biological computer model of bone regeneration to explain scaffold-supported bone healing and investigate the distinct roles of implant structure and bone grafting on the regeneration process within a scaffold. In addition, Marco et al. (Tatullo et al., 2019a; Tatullo et al., 2019b) considered that 3D printing implant doped or coated with novel biomaterials like Phosphorene or Borophene could be a useful strategy to improve the therapeutic effect of osteogenesis. And the *in vivo* studies have revealed that the coated implants worked effectively on post-oncological bone defects (Yang et al., 2018). These studies provide a theoretical basis for the design and optimization of bone regeneration implants, making it possible to reconstruct mandibular with 3D implants combined with bone graft materials.

In this study, we proposed a novel 3D titanium lattice-like implant based on simulation model, which is designed and optimized by a biomechanical/mechanobiological approach, and the implant can be filled with bone graft materials to promote bone regeneration for mandibular injuries repair. There are some special aspects of lattice-like implant compared to existing 3D titanium plate/mesh implants. Firstly, the clinical strategy of the lattice-like implant is novel. The existing 3D titanium plate/mesh implants are designed for mandibular contour restoration without considering bone regeneration. While the lattice-like implant not only restores maxillofacial contour, but also the bone regeneration by filling bone graft materials and ultimately to achieve occlusal reconstruction. Secondly, design and optimization of lattice-like implants based on biomechanical and mechanobiology methods. Because of the filling of bone graft materials, the design and optimize of lattice-like implant should not only consider its biomechanical properties, but also the bone remodelling process of the bone graft material under mechanical stimulation. That is, the implant should have sufficient biomechanical properties to avoid mechanical failure and the bone graft materials should be in the strain adaptation ranges of remodeling to induce bone regeneration. Thirdly, novel structural in lattice-like implant design. The lattice-like implant contains internal trabecular-like structure to simulate cancellous bone and the external grid-like structure to simulate cortical bone function. The regular dodecahedron structure has been suggested as representative of 3D organization of trabecular bone (Gramanzini et al., 2016; Ben-Zvi et al., 2017; Gao et al., 2019), to mimic the same biomechanical environment of the mandible, we chose a dodecahedral cellular unit presenting an analogous texturing to obtain the trabecular-like component of the implant. And the pore diameter of implant was selected to be 5 mm to facilitate the filling of bone graft materials which diameter is generally 1∼2 mm and the maintaining adequate blood supply. Figure 1D illustrates the overall view of local orientation of the mandible which is taken from reference (Apicella et al., 2010). The arrow indicates the direction of maximum stiffness concerning the occlusal plane and the primary struts have been oriented according to this natural biomechanical requirement in the lattice-like implant.

Mechanical stimulations at an appropriate magnitude are known to promote tissue mineralization and new bone formation from Frost’s “Mechanostat” theory (Frost, 2004). The finite element method was utilized to obtain detailed stress, strain and displacement distributions, which determines whether the implant needs optimization. The FEA and frequency analysis results of the original implant indicated that the implant probably has partial struts fracture due to the high region of stress concentration and only 35.79% bone graft materials in the strain adaptation ranges of remodeling during the G-T period. Therefore, the stress concentration area has been optimized based on biomechanical and mechanobiological principles by tuning the local struts diameters of the original implant. Encouraging results have been obtained from the FEA, and frequency analysis results of optimized implant after several iterations demonstrated that the implant has sufficient mechanical strength and the bone graft materials have favourable biomechanical stimulation for bone defect regeneration. It is noteworthy that the maximum von-Mises stress values of the optimized implant are 697.35 MPa in the G-T period while only stress values of eight nodes exceed 500 MPa; we thoroughly considered that this is probably caused by the finite element mesh distortion of struts edge and that is the reason why frequency analysis approach was adopted to statistics the value of each node. Therefore, the iteration of topology optimization did not continue. Through the iterations based on biomechanical and mechanobiological principles, the maximum von-Mises stress of implant was decreased by 43.14% on average, and the favourable strain of bone graft increased from 35.79% to 93.36% in the early critical regeneration stages. These results demonstrated the effectiveness and necessity of the iterative optimization process based on biomechanical/mechanobiological approach.

Although the validity and feasibility of the working framework are verified in this study, and has been adopted by clinical institutions for clinical case implementation (unpublished data), its limitations still need to be noted. Whether the framework has enough repeatability and controllability is one of the main not yet verified limitations of this study. As a custom implantable device, clinical scenarios are complex and changeable, but we have only completed the design and preparation process of a few cases so far. It is unclear whether the framework will be robust enough to deal with more complicated case scenarios in the future, when clinical cases increase significantly. Therefore, more clinical studies or animal experiments need to be completed to verify its further clinical appropriateness.

## Conclusion

In summary, this study proposed a novel 3D titanium lattice-like implant for mandibular injuries based on simulation model, which is designed and optimized by a biomechanical/mechanobiological approach, and the working framework for optimal design and preparation processes of the 3D titanium lattice-like implant has been validated. The results illustrated that a biomechanical/mechanobiological optimized 3D lattice-like implant filled with bone graft materials possesses sufficient mechanical strength to avoid mechanical failure and providing necessary biomechanical stimulus to promote bone regeneration. This study is expected to provide a scientific and feasible clinical solution for repairing large mandibular injuries and offers important insights into future design criteria for mandibular reconstruction.

## Data Availability

The original contributions presented in the study are included in the article/Supplementary Material, further inquiries can be directed to the corresponding author.
